# Thermal Wavelength Measurement of Nanofluid in an Optical-Fiber Thermal Wave Cavity Technique to Determine the Thermal Diffusivity

**DOI:** 10.1155/2018/9458952

**Published:** 2018-03-01

**Authors:** Monir Noroozi, Bijan Mohammadi, Shahidan Radiman, Azmi Zakaria

**Affiliations:** ^1^School of Mechanical Engineering, Iran University of Science and Technology, Narmak, Tehran 16846, Iran; ^2^School of Applied Physics, Faculty of Science and Technology, Universiti Kebangsaan Malaysia (UKM), 43600 Bangi, Selangor, Malaysia; ^3^Department of Physics, Faculty of Science, Universiti Putra Malaysia (UPM), 43400 Serdang, Selangor, Malaysia

## Abstract

The application of optical-fiber thermal wave cavity (OF-TWC) technique was investigated to measure the thermal diffusivity of Ag nanofluids. The thermal diffusivity was obtained by measuring the thermal wavelength of sample in a cavity scan mode. The spherical Ag nanoparticles samples were prepared at various sizes using the microwave method. Applying the thermal wavelength measurement in a flexible OF-TWC technique requires only two experimental data sets. It can be used to estimate thermal diffusivity of a small amount of liquid samples (0.3 ml) in a brief period. UV-Vis spectroscopy and transmission electron microscopy were used to measure the characterization of the Ag nanoparticles. The thermal diffusivity of distilled water, glycerol, and two different types of cooking oil was measured and has an excellent agreement with the reported results in the literature (difference of only 0.3%–2.4%). The nanofluids showed that the highest value of thermal diffusivity was achieved for smaller sized nanoparticles. The results of this method confirmed that the thermal wavelength measurement method using the OF-TWC technique had potential as a tool to measure the thermal diffusivity of nanofluids with different variables such as the size, shape, and concentration of the nanoparticles.

## 1. Introduction

In photopyroelectric (PPE) methods [1, 2], the thermal wave cavity (TWC) technique has been extensively applied for measuring the thermal properties of various types of liquids [3]. The TWC technique allows thermal waves to be detected by cavity length scan, instead of on frequency modulation. The major advantage of cavity length scan is the fixed noise bandwidth of the system that has a high signal-to-noise ratio (SNR) in thermally thick case [4]. The TWC technique is based on the conversion of part of or all of the optical energy into heat by a metallic light absorber, where this process within a metal can give rise to a pyroelectric (PE) signal, by a pyroelectric sensor, polyvinylidene difluoride (PVDF), which is in contact with the sample. The conventional TWC technique has been modified by replacing the metallic foil with an optical fiber which acts to channel light and to generate thermal waves (TWs) at the metalized fiber tip. This has been a significant instrumental advance in the field of PPE techniques [5]. Unlike conventional TWC techniques, the optical-fiber thermal wave cavity (OF-TWC) technique does not require a tube and metal foil. This makes it possible to be used in small volume detection, with a simple arrangement of the TWs generator-detector, and makes the whole system more signified with measurable improvement in signal stability and SNR [6].

Different schemes have been used for thermal diffusivity evaluation via the TWC theory, including direct and indirect evaluation. In the direct evaluation, the thermal diffusivity is obtained from the fitting of the phase (and in-phase) and amplitude (and quadrature) of the signal as a function of cavity length [7]. The thermal diffusivity of liquid sample is frequently obtained through fitting the amplitude and phase of the PE signal in a cavity scan. However, it is hard to fit the experimental data of the sample with a low thermal diffusivity [8]. Other methods involved the indirect evaluation of the thermal wavelength from the phase of the signal via the cavity lengths, where the thermal wave has its first two extrema [9]. The thermal diffusivity of the sample is subsequently calculated from half of the thermal wavelength. The OF-TWC method of the PPE techniques is a simple and suitable design for measuring the thermal diffusivity of liquid, due to the small volume of the detection cell and ease in performance of the measurement. However, this configuration may have some limitation to measure thermal diffusivity, especially at large cavity lengths [10]. OF-TWC technique can be applied to determine the thermal wavelength of nanofluid using only two involved cavity lengths. The phase difference is equal to half of the thermal wavelength, and the thermal diffusivity of nanofluid can easily be measured in a short measurement interval, which leads to more accurate results than that of the amplitude and phase fitting method. The advantage of this measurement is its high sensitivity in the minimum measurement time required (~3 min) of a small volume of sample (which makes it suitable for nanofluids). In this work, the feasibility of measuring the thermal wavelength in the OF-TWC technique was examined.

Thermal research in nanofluids has increased in the last years, especially due to the higher thermal properties of the nanofluids and their various applications including photothermal therapy and creating the next-generation thermofluids [11, 12]. Generally, microwave irradiation (MI) technology is a viable avenue for the greener synthesis of nanomaterials and metallic nanoparticles, because it is clean, rapid, and simple to use [13, 14]. Recently, some techniques have been developed for measuring and elucidating the thermal conductivity and thermal diffusivity of nanofluids [15–17], as well as observing the influence of particle nanostructures on their thermal properties. Thermal properties of nanofluids are typically measured using thermal lens spectrometry, hot wire, open photoacoustic cells, and PPE techniques [18–21].

In this work, the thermal diffusivity of a small amount (~0.2 ml) Ag nanofluid was measured using the thermal wavelength method of the OF-TWC technique. Solutions of Ag NPs in water and in the presence of polyvinylpyrrolidone (PVP) were prepared under different MI times, from 20 to 100 s. The study of the thermal diffusivity of the Ag nanofluids as a function of particle size (16–53 nm) was also carried out. Before measuring the thermal diffusivity of the Ag nanofluids, the thermal wavelength method was applied to measure the thermal diffusivities of some liquids, such as distilled water, glycerol, and two different types of cooking oil. The thermal diffusivity values obtained from this method confirmed the capability of the thermal wavelength method of the OF-TWC technique for measuring the thermal diffusivity in nanofluids, within a small experimental error of 10^−5^ cm^2^/s. The obtained nanoparticles were also characterized by UV-Vis spectroscopy and transmission electron microscopy (TEM).

## 2. Theoretical Background

The TWC configuration of the PE signal of a liquid, in a one-dimensional case, is given by [22]

(1) Vf,lm=C×e−σmlm1−RsmRmpe−2σmlmRij=1−bij1+bij;bij=kjkiαiαj1/2.

In (1), *ij* represents the TWs generator (*s*), liquid medium (*m*), and PE detector (*p*) layers of the PE cell, respectively. *C* is an instrumental factor, *f* is the frequency of light modulated, *k* is the thermal conductivity, *α* is thermal diffusivity, *l*_*i*_ is the thickness of the layer *i*, *σ*_*j*_ is the complex TWs diffusion coefficient *σ*_*j*_ = (1 + *i*)*a*_*j*_, *a* is the reciprocal of the thermal diffusion length *μ*, *a* = 1/*μ*, and *μ* = (*α*/*πf*)^1/2^. In the thermally thick regime for both the detector and the sample (*μ*_*i*_ ≪ *l*_*i*_), then the term *e*^−2*σ*_1_*l*_1_^ ≈ 0, (1) can be written more simply as

(2) $$\begin{array}{c} V\left(\;f,l_{m}\;\right)=Const\left(\;f,l_{m}\;\right)\times e^{-\sigma_{m}l_{m}} \end{array}$$

⟹

(2a) ReVf,lm=IP=Constf,lme−amlmcos⁡amlm

(2b) ImVf,lm=Constf,lme−amlmsin⁡amlm.

Equation (2) shows that the PE signal depends on the modulation frequency/and cavity length *L*. In (2), there are standing wave patterns in both the in-phase and quadrature of the PE signal as the real (see 2a) and the imaginary (see 2b) parts of the PE signal. These real and imaginary parts of the signal are like the pressure waves in the acoustic waves in the music tube. However, the damped nature of thermal waves makes them exponentially decay within the cavity from source to detector. From the voltage of the PE signal, the amplitude and phase of the PE signal can be written, respectively, as

(3a) Vf,l=e−πf/α1/2l

(3b) φf,l=−πfα1/2l.

The logarithm of the amplitude and phase of signal versus the cavity length have a linear behavior. The thermal diffusivity can be obtained from the slope fitting of the amplitude and the phase versus the cavity length. On the other hand, from the thermal wave phase lag, the thermal diffusivity measurement can be also obtained by the measurement of the thermal wavelength which is defined as [9]

(4) $$\begin{array}{c} \lambda =2\sqrt{\frac{\pi \alpha }{f}}. \end{array}$$

Hence, the sample's thermal diffusivity can be obtained as

(5) $$\begin{array}{c} \alpha_{l}={\left(\;\frac{\lambda }{2}\;\right)}^{2}\frac{f}{\pi }. \end{array}$$

The procedure involves the phase of the PE signal at two different cavity lengths in which the phase difference is equivalent to one-half of the wavelength, where the phase takes on the values (−*π*) and 0. The thermal diffusivity of the sample was obtained directly by measuring the cavity related to the half thermal wavelength of the phase. The experimental methodology consisted of applying the thermal wavelength, for measuring the thermal diffusivity of the sample. The half thermal wavelength, *λ*/2, was directly obtained by measuring the distance between the phase equaling −*π* and when the phase equaled zero. The experimental error was obtained by using the standard formula for error propagation in *α* = (*λ*_TM_/2)^2^*f*/*π*, where Δ*α* was calculated by Δ*α*/*α* = 2((Δ*λ*/2)/(*λ*/2)) + Δ*f*/*f*.

## 3. Materials and Methods

### 3.1. Preparation of the Silver Nanofluids

For preparing the Ag nanofluids samples silver nitrate (AgNO_3_-Merck, Darmstadt, Germany, 99.98%, 4.35 g/cm^3^) was used as the source of silver NPs and PVP (MW–29000, K25, 1.2 g/cm^3^, Aldrich Chemistry) was used as a stabilizer for the Ag NPs fabrication. The PVP solution was prepared by dissolving 1 g PVP powder in 50 ml deionized water. The solution was magnetically stirred for 30 min. 0.5 g AgNO_3_ was added to the PVP solution to prepare the Ag nanofluid samples. A microwave oven (Panasonic, 1100 W, 2.45 GHz, NN-K574MF, multimode) was used as an environmentally effective tool in performing chemical changes in minutes, as per a procedure reported in our previous paper [21]. The solutions were then irradiated with MI at 20, 40, 60, and 100 s to produce various nanoparticle sizes (S1–S4). In this process, the MI reaction and the source of silver caused the Ag ions to reduce to Ag atoms. Fast nucleation during the MI led to the formation of high number of nanoparticles with more uniform size distributions. The particle size, shape/morphology, and distribution of the nanoparticles were determined by transmission electron microscopy. The particles in the solution were uniform. The absorption spectra of the samples were measured using a UV-Vis spectrophotometer (Shimadzu-UV1650PC).

### 3.2. Experimental Procedures

**Figure 1:**
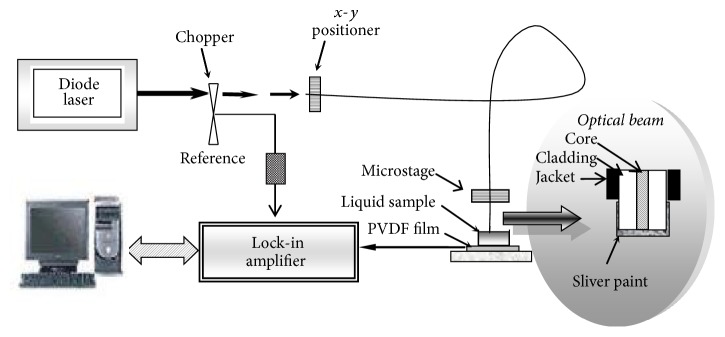
Schematic diagram of OF-TWC technique.

The OF-TWC setup has been developed as a technique for measuring the thermal diffusivity of liquids in a small volume by using cavity length scanning [10]. Briefly, as shown in Figure 1, the light modulated beam from the diode pumped solid state laser (200 milliwatt-532 nm wavelength) was transmitted through the inlet of the polymer fiber (RS 368-047, 2.25 mm outer diameter and 1.0 mm core diameter). The metalized optical fiber was used as the light transfer agent and also as the thermal wave generator. The fiber tip was coated with a very thin matte black paint as an efficient light-to-heat converter and the thin layer of the silver conductive paint acted as a thermal wave generator. This fiber tip was fixed to a micropositioner for the purpose of varying the cavity length *L*, in 10 *μ*m step resolutions, with respect to the PE detector (a PVDF film, 52 *μ*m thick). The PE voltage signal generated in the sensor was analyzed by the lock-in amplifier and the frequency was fixed at 6.73 Hz.

**Figure 2:**
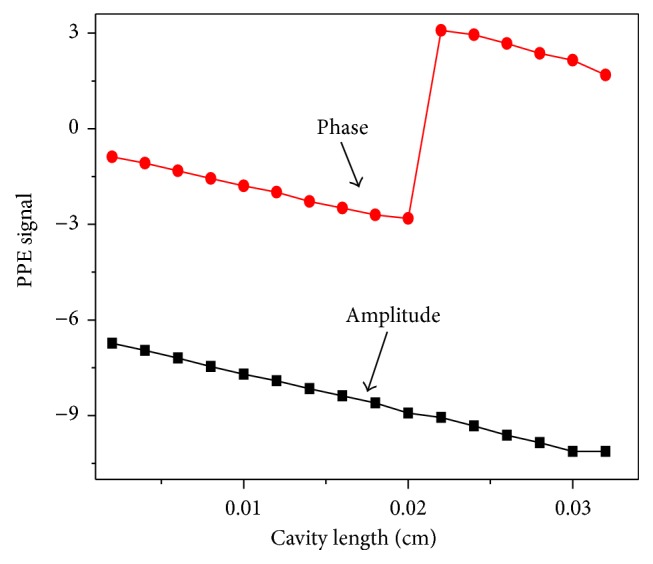
Phase (radian) and logarithm of amplitude of the PE signal as a function of the cavity length. The jump in-phase shows a discontinuity between −*π* and *π*.

All the measurements were made at room temperature (25°C). The phase discontinuity was created by choosing a given instrumental reference phase lags. The Origin 7.5 program was used to plot the graph of phase versus cavity length. In a cavity length scanning, based on the logarithm of the amplitude one may note that there was a continuous linear behavior in the PE signal. A similar behavior of the PE phase was obtained at the same modulated frequency (6.73 Hz). However, a jump was seen in the phase plot, which occurred when the phase took on the (−*π*), where the phase jumped from (−*π*) to (*π*) after one full thermal wave cycle (as shown in Figure 2).

## 4. Results and Discussion

### 4.1. Nanoparticle Characterization

**Figure 3:**
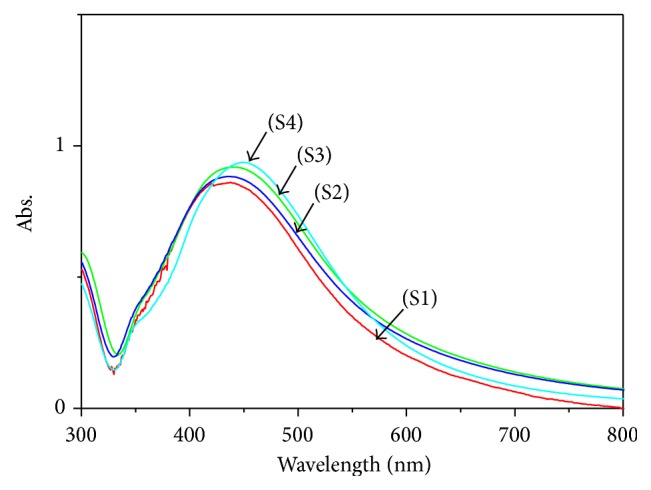
Optical absorption spectra of fluids containing Ag nanoparticles prepared at different MI times.

**Figure 4:**
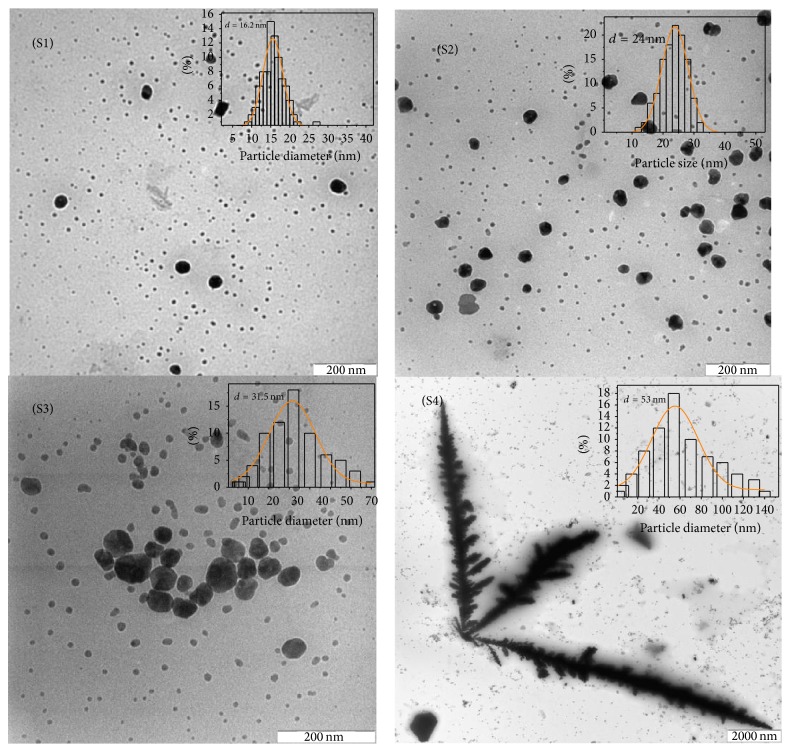
The TEM images and size distributions of the four particle diameters obtained at different MI times, (S1) 16, (S2) 24, (S3) 31, and (S4) 53 nm.

Figure 3 shows the UV-Vis absorption spectra of the Ag nanofluids under different MI times 20 s (S1), 40 s (S2), 60 s (S3), and 100 s (S4) in deionized water in the presence of PVP. In these spectra, the surface plasmon resonance (SPR) band had peaks at 430–450 nm, which was generally assigned to the absorption peak of the spherical Ag nanoparticles [12]; this was confirmed by the TEM results. From Figure 3, as the irradiation time increased, the red-shift was observed in UV–visible spectrum. This red-shift was related to an increase in the size of particles and also agglomeration of silver nanocrystals [12] due to the rapid heating and high efficiency of the MI [13]. The intensity of the SPR peak also showed a small increment; this phenomenon is related to an increase in the seed concentration [14], when irradiation time increased. Therefore, the nucleation processes of the silver NPs have continued and resulted in an increase in the concentration of Ag NPs. However, longer MI time promoted particle aggregation and formation of the Ag dendrites, as can be seen from the TEM images in Figure 4.

Figure 4 shows the TEM images and the size distribution of the Ag nanoparticles obtained with increasing the MI irradiation time from 20 to 100 s. As seen in Figure 4, the mean particle size of the Ag NPs increased from 16 to 53 nm, when irradiation time increased as was expected. The result showed that there was an apparent increase in concentration and the amount of many small NPs that appeared after irradiation. However, the formation of silver trees was observed (when the irradiation period was increased to 100 s). Figure 4(S4), the TEM image shows that the large amount of the particles is near to spherical in shape with an average particle size of 53 nm, and there is small amount of the Ag dendrite which had the length 3–6 *μ*m and the width of 100–200 nm. As the sphere grew, the spherical morphology became unstable and its shape became perturbed. The solid shape began to express the preferred growth directions of the crystal. Consequently, the rate of nucleation and growth of crystals caused the formation of the Ag dendrites structure [23].

### 4.2. Thermal Diffusivity Measurements

**Figure 5:**
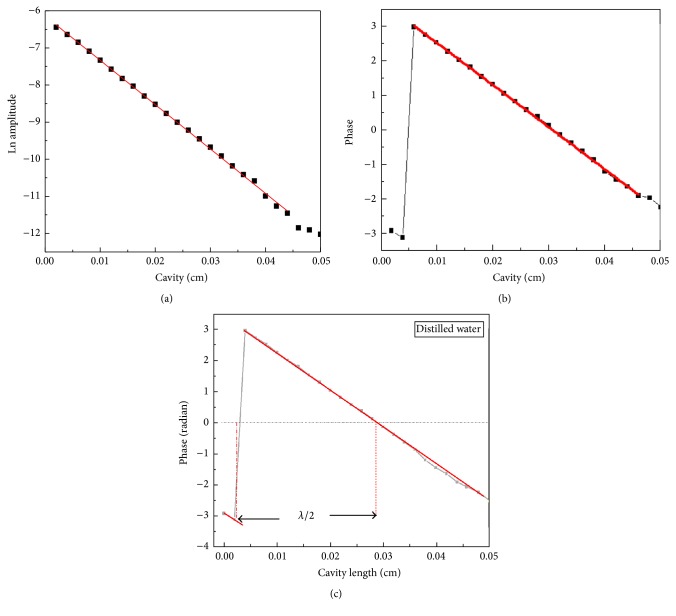
The typical behavior of the Ln amplitude and the phase as a function of the cavity length for the distilled water, at 6.73 Hz, (a) the plots of logarithmic amplitude and (b) the phase versus the cavity length, and (c) the plot of phase versus the cavity length; the half thermal wavelength was directly calculated by obtaining the cavity length between two experimental data sets; the phase equals (−*π*) and the phase equals (0).

Before measuring the thermal diffusivity of Ag nanofluids, the experimental system was calibrated by measuring the thermal diffusivity of distilled water as the reference fluid using the thermal wavelength method.  Figure 5 shows (a) the linear plot of Ln amplitude and (b) the phase versus relative cavity length *L*, respectively, and (c) the typical behavior of the phase as a function of the cavity length for distilled water, at 6.73 Hz. As shown in Figure 5(c), the half thermal wavelength was directly calculated by obtaining the cavity length between two experimental data sets, the phase equals (−*π*), and the phase equals (0). The half thermal wavelength of water in this frequency was 0.026 cm. By applying (5), the thermal diffusivity of distilled water obtained from the thermal wavelength measurement was *α* = (1.450 ± 0.110) × 10^−3^ cm^2^/s; this result agrees very well with the values reported in the literature (1.445 × 10^−3^ cm^2^/s) [4].

**Figure 6:**
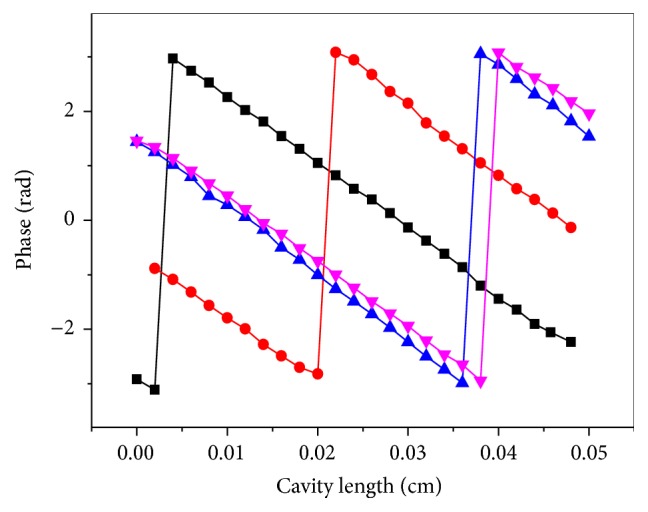
Variation of the PE signal phase as a function of cavity length for different instrumental reference phase. Plots corresponding to different instrumental phase reference phase are shifted from each other.

As shown in Figure 6, the phase jump could be changed by choosing different instrumental reference phase lags. It was evident from Figure 6 that the phase deviated from the expected linear behavior at long cavity lengths. It was important to choose the optimal value of cavity length for the thermal wavelength measurement by means of the present methodology. The smaller the cavity length, the better the output phase of the signals [24]. Thus, the instrumental reference phase lag was favored in using the thermal wavelength methodology as the jump of the phase shifted to a small cavity length.

**Figure 7:**
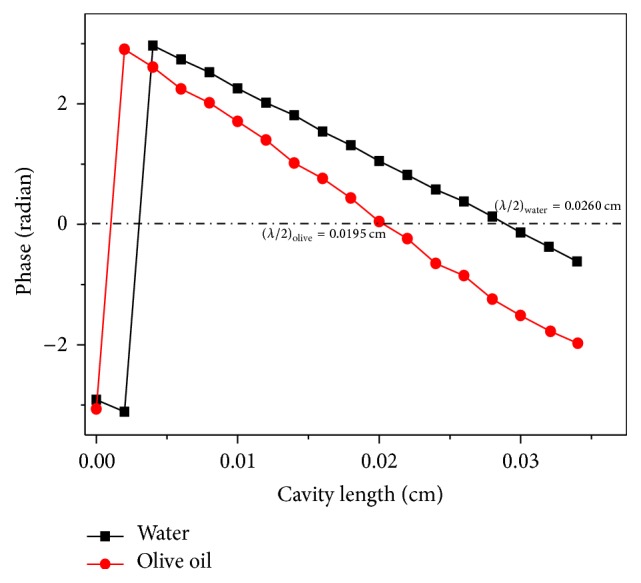
PE signal as a function of the cavity length for phase discontinuities for distilled water and olive oil.

To complete the analysis and to test the reliability of this methodology, the thermal diffusivities of glycerol and two cooking oils were measured by the thermal wavelength method.  Figure 7 shows the plot phase of distilled water and olive oil versus the cavity length at the same modulation frequency and the same reference phase lag. As shown in Figure 7, the half thermal wavelength was directly obtained by measuring the relative cavity length between the first phase discontinuities (phase equals −*π*) and the phase equals (0). It can be observed that the thermal wavelengths of water and olive oil are 0.0260 (cm) and 0.0195 (cm), respectively; it was possible to calculate their thermal diffusivities by using (5), by means of this methodology. The thermal diffusivity of glycerol, sunflower oil, and olive oil, obtained from the thermal wavelength method, is (0.914 ± 0.045) × 10^−3^ cm^2^/s, (1.111 ± 0.045) × 10^−3^ cm^2^/s, and (0.818 ± 0.040) × 10^−3^ cm^2^/s, respectively, which differs by only about 0.3%–2.4% with values described in the literature [4, 9].

**Figure 8:**
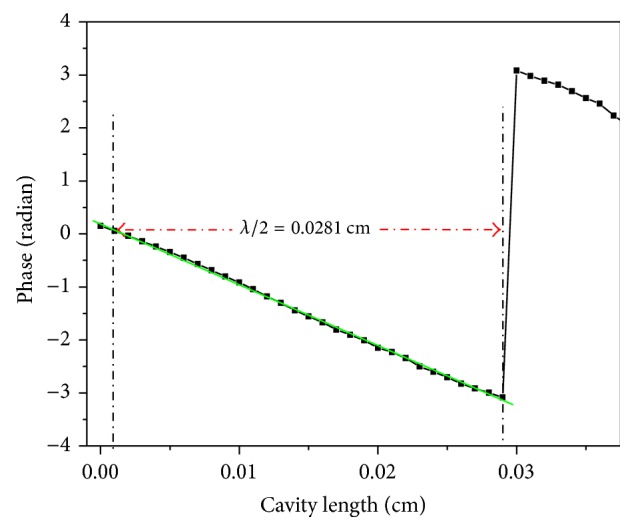
Typical behavior of the phase of PE signal as a function of cavity length for Ag nanofluid (S1) at 6.73 Hz.

This methodology is simpler than the fitting methodology, because only a minimum time (~3 min) was required, and only two relative cavity lengths were involved. However, the accuracy of the thermal diffusivity measurements in this method was directly related to the accuracy of the thermal wavelength measurements [9]. The small experimental error, 10^−5^ cm^2^/s, indicated the accuracy of this technique in measuring the liquid thermal diffusivity of small volume samples. In order to evaluate the applicability of this method, the thermal diffusivity of the Ag nanofluids was obtained from the thermal wavelength measurements. The typical behavior of the PE signal phase as a function of cavity length for the Ag nanofluid (S1) at 6.73 Hz is shown in Figure 8.

The half thermal wavelength value was 0.0281 cm, between two relative cavity lengths, the phase equals (−*π*), and the phase equals (0), using (5). The thermal diffusivity value obtained from thermal wavelength measurement was (1.691 ± 0.060) × 10^−3^ cm^2^/s. This indicated that the addition of nanoparticles to the base fluid led to a significant increase in the thermal diffusivity.

This was because the thermal diffusivity of nanofluid depended on some parameters, such as nanoparticles material, concentration of particles, size, base fluid, and surfactant. The thermal diffusivity of Ag nanofluid was improved by using Ag NPs with high thermal properties [25]. The resulting thermal diffusivity values of Ag nanofluids in different irradiation times were sorted in Table 1.

**Figure 9:**
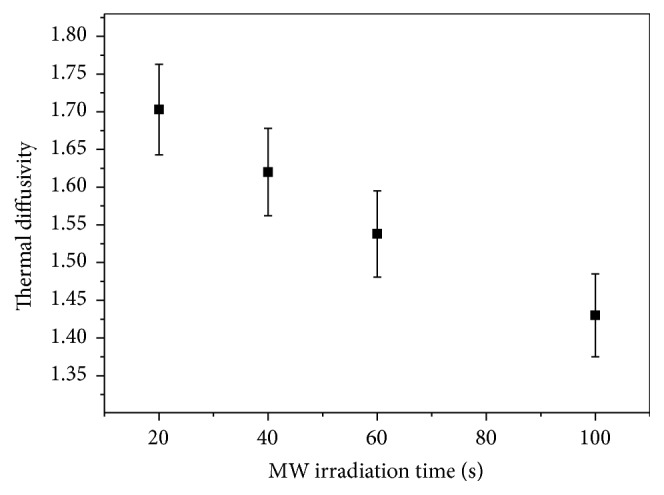
Thermal diffusivity Ag nanofluids with varying MI time.

Figure 9 shows the thermal diffusivity data of the Ag nanofluids as a function of MI times. As shown in Figure 9, when the MI time increased, the thermal diffusivity of the nanofluids decreased. It was observed that, by increasing the irradiation time, the aggregation of NPs could be increased, leading to change in the structure size and shape of particles. Microwave irradiation plays an important role in the aggregation process; higher irradiation times make aggregation process faster. On the other hand, at low irradiation time, due to less number of the Ag NPs in the solution, the aggregation of the nanoparticles cannot be stated. This was because smaller particles have a higher effective surface to volume ratio of the nanoparticles [21], thus increasing the thermal properties of the nanofluids. Similar results have been reported in the literature for the thermal diffusivity of metal nanofluids, as measured using the photoacoustic technique thermal lens spectrometry, and the hot wire method [18–21]. A maximum enhancement of thermal diffusivity by about 17% was found for Ag nanofluid prepared under only 20 s irradiation times. It has a similar increasing trend to other results which obtained Ag/water nanofluid by using a laser flash photoacoustic method [26]. The results [16] also recorded the increase in the thermal diffusivity as the particle size decreased. However, there was no enhancement in the thermal diffusivity of S4 with larger-diameter (55 nm) concentrated nanofluids, as compared to other nanofluids. These results are also possibly attributable to the rapid particle clustering and forming the aggregates of NPs [27]. The data obtained indicated the successful use of the OF-TWC experimental setup for thermal characterization of nanofluids using the thermal wavelength method with high accuracy and minimum measurement times (about 3 min).

## 5. Conclusions

The accuracy of the OF-TWC technique was investigated to measure the thermal diffusivity of Ag nanofluids by measuring the thermal wavelength of nanofluid. The nanofluids ranged in size, from 16 to 53 nm, and were prepared by a MI method. The thermal diffusivity of the Ag nanofluids changed inversely with the particle size, which was a similar behavior in thermal properties that were reported by other techniques, such as thermal lens and hot wire methods. A maximum enhancement of thermal diffusivity by about 17% was found for Ag nanofluid prepared under only 20 s irradiation time The study showed that the OF-TWC technique was suitable for measuring the thermal diffusivity of Ag nanofluids by using a small volume of the nanofluid sample (0.2 ml) under minimum measurement (about 3 min) times.

## Figures and Tables

**Table 1:** Summarized results for thermal diffusivity of Ag nanofluids with varying MI time at different particle sizes.

Sample	Irradiation time (s)	Particle size (nm)	*λ*/2 (cm)	*α* (10^−3^ cm^2^/s)
S1	20	16.2	0.0281 ± 0.0005	1.691 ± 0.060
S2	40	24	0.0275 ± 0.0005	1.620 ± 0.058
S3	60	31.5	0.0268 ± 0.0005	1.538 ± 0.057
S4	100	53	0.0258 ± 0.0005	1.430 ± 0.055
